# A Rare Case of Lateral Forefoot Pain: Plantar Adventitious Bursitis

**DOI:** 10.7759/cureus.9011

**Published:** 2020-07-05

**Authors:** Min Cheol Chang, Wei-Ting Wu, Ke-Vin Chang

**Affiliations:** 1 Physical Medicine and Rehabilitation, College of Medicine, Yeungnam University, Daegu, KOR; 2 Physical Medicine and Rehabilitation, National Taiwan University Hospital Bei-Hu Branch, Taipei, TWN

**Keywords:** pain, forefoot, ultrasonography, plantar adventitious bursitis

## Abstract

Forefoot pain is a common musculoskeletal complaint but is rarely caused by plantar adventitious bursitis. A 27-year-old female had right lateral forefoot pain for three weeks and was referred for an ultrasound examination, revealing an anechoic mass on top of the flexor digiti minimi brevis tendon. Two weeks after oral medication and a prescription of rocker-bottom shoes, her pain totally disappeared. In conclusion, ultrasound is helpful in differentiating various causes of forefoot pain, which, in this case, facilitated the detection and management of plantar adventitious bursitis.

## Introduction

The feet transmit the body weight to the ground and maintain the equilibrium of posture during locomotion. During the foot strike, the feet act as a shock absorber and then become the lever for push-off [1]. Since the feet bear the weight of the entire body, the pertinent muscles, tendons, or ligaments are prone to injury. Foot disorders hinder ambulation and worsen patients’ quality of life [2]. Tendon sprain, stress fracture, arthritis, interdigital neuroma [3], and intrinsic muscle disorders [4] can elicit foot pain. However, little is known about the pain caused by plantar adventitious bursitis [5]. In this regard, we would like to report a case with lateral forefoot pain caused by plantar adventitious bursitis and how useful ultrasound (US) was for diagnosis.

## Case presentation

A 27-year-old female visited our clinics because of pain in the right lateral and plantar forefoot for three weeks. The pain had an insidious onset and aggravated while walking. She did not participate in any sports. However, due to her job (a waiter), she had to walk and stand for a long period during work hours. Her body weight was 43 kilograms, and no pre-existing musculoskeletal diseases or trauma at her feet was mentioned. During the physical examination, the pain was elicited upon dorsiflexion of the right fifth toe. There was no motor or sensory deficit over the affected area. Tenderness was also observed over the plantar aspect of the right fifth metatarsal bone whereas no local swelling was palpated. The plain radiograph was normal.

**Figure 1:**
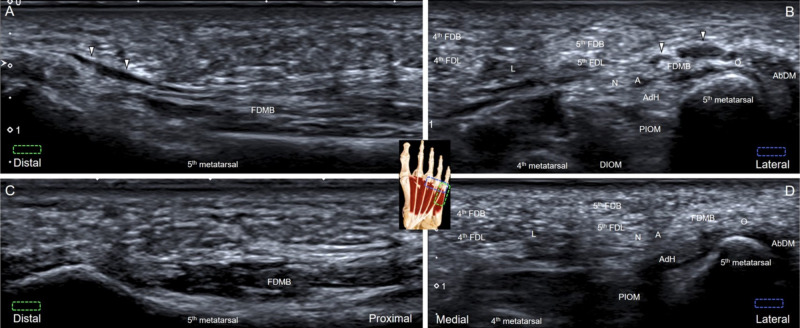
Plantar adventitious bursitis The anechoic mass was noted superficial to the flexor digiti minimi brevis in its long-axis (A) and short-axis view (B). No similar lesion can be identified in the long-axis (C) and short-axis views (D) at the contralateral, asymptomatic side. White arrowheads: plantar adventitious bursitis FDB: flexor digitorum brevis; FDL: flexor digitorum longus; AbDM: abductor digiti minimi; PIOM: plantar interosseous muscle; DIOM: dorsal interosseous muscle; N: nerve; A: artery; L: lumbrical muscle; O: opponens digiti minimi; AdH: transverse head of adductor hallucis

She was referred for an ultrasound examination. The linear transducer was placed on the plantar surface in line with the fifth metatarsal bone. When tracing the flexor digiti minimi brevis (FDMB) tendon from its metatarsal origin to the insertion of the fifth proximal phalanx, we identified a non-compressible anechoic mass without increased intralesional vascularity (Figure 1A). In the short-axis view, the lesion was confirmed to be at the bottom of the subcutaneous layer and between the tendons of the FDMB and the abductor digiti minimi muscle (Figure 1B). Sonopalpation over that region reproduced exactly the same pain as the pain she experienced during walking. No similar lesion was identified at the contralateral side (Figures 1C-1D). Plantar adventitious bursitis was diagnosed. Two weeks after oral medication with non-steroid anti-inflammatory drugs and a prescription of rocker-bottom shoes, her pain totally subsided.

## Discussion

When the feet are loaded on the ground, mechanical energy is stored and then released by the intrinsic muscles, tendons [6], and soft tissues. This function is important for acceleration, deceleration, and change of directions [6], whereas the friction generates shear forces between the plantar adventitious bursa and the aforementioned foot structure [7]. Plantar adventitious bursitis is characterized by myxomatous degeneration and inflammation of superficial fibrous connective tissue [7]. In comparison to the synovial bursa located among muscles, tendons, ligaments, or bones, the inner surface of the adventitious bursa is not lined by synovium [5]. Plantar adventitious bursitis usually occurs at the first and fifth metatarsal heads [4] and should be distinguished from other disorders, including stress fractures, plantar plate ruptures, arthritis, intermetatarsal bursitis, Morton neuromas, and tenosynovitis. All lesions mentioned above are still difficult to diagnose through physical examinations because the related structures are approximated and overlapped together at the plantar area.

US is a useful tool for determining the location and extent of the foot lesions [8-9]. US is widely used in musculoskeletal medicine because it can provide real-time imaging and dynamic assessments [4,9]. Scanning the whole metatarsal bones and adjacent joints can help identify stress fractures, plantar plate ruptures [10], and joint arthritis/synovitis. The transducer can be placed along the anterior transverse foot arch for the investigation of Morton neuromas and intermetatarsal bursitis [11].

Tenosynovitis of the flexor digitorum longus (FDL), flexor digitorum brevis (FDB), and FDMB can affect normal ambulation. The investigator can place the transducer parallel to the lateral aspect of the fifth metatarsal bone and track the FDMB to its insertion on the proximal phalanx. Herewith, if the transducer is placed at the medial aspect of the fifth metatarsal bone with its proximal end pointing to the medial foot arc, the FDB and FDL tendons can be clearly visualized along their long axis. The transducer can be redirected to the transverse plane to visualize the hypoechoic fourth lumbrical muscles located beside the FDL tendon.

Some clinical pearls are worth sharing regarding investigation for patients with lateral forefoot pain. First, the investigators should be aware of the fact that the lesions over the forefoot may be difficult to visualize because of the thick keratin layer, which attenuates the US signals [4]. Second, plantar adventitious bursitis occurs at the subcutaneous layer, whereas the underlying toe flexor tendons and intermetatarsal bursa should not be affected.

## Conclusions

The causes of lateral forefoot pain are miscellaneous and difficult to diagnose simply by history taking and physical findings. Static and dynamic US examinations are helpful for differential diagnosis. Since plantar adventitious bursitis is not rare but commonly overlooked, its related foot discomfort is likely to persist if a correct diagnosis is not made. In short, for patients with refractory forefoot pain, we recommend US to be the prioritized imaging tool to investigate its underlying pathology.
